# Federated Reinforcement Learning Based AANs with LEO Satellites and UAVs

**DOI:** 10.3390/s21238111

**Published:** 2021-12-04

**Authors:** Seungho Yoo, Woonghee Lee

**Affiliations:** 1 School of Electrical Engineering, Korea University, Seoul 02841, Korea; pen0423@korea.ac.kr; 2 Division of IT Convergence Engineering, Hansung University, Seoul 02876, Korea

**Keywords:** aerial access network, federated reinforcement learning, low-Earth orbit satellites, pseudo-satellites, non-terrestrial network

## Abstract

Supported by the advances in rocket technology, companies like SpaceX and Amazon competitively have entered the satellite Internet business. These companies said that they could provide Internet service sufficiently to users using their communication resources. However, the Internet service might not be provided in densely populated areas, as the satellites coverage is broad but its resource capacity is limited. To offload the traffic of the densely populated area, we present an adaptable aerial access network (AAN), composed of low-Earth orbit (LEO) satellites and federated reinforcement learning (FRL)-enabled unmanned aerial vehicles (UAVs). Using the proposed system, UAVs could operate with relatively low computation resources than centralized coverage management systems. Furthermore, by utilizing FRL, the system could continuously learn from various environments and perform better with the longer operation times. Based on our proposed design, we implemented FRL, constructed the UAV-aided AAN simulator, and evaluated the proposed system. Base on the evaluation result, we validated that the FRL enabled UAV-aided AAN could operate efficiently in densely populated areas where the satellites cannot provide sufficient Internet services, which improves network performances. In the evaluations, our proposed AAN system provided about 3.25 times more communication resources and had 5.1% lower latency than the satellite-only AAN.

## 1. Introduction

While cellular networks have been evolved continuously to 6th generation (6G), the terrestrial network was the major component of the cellular network. To construct the terrestrial network, ground-based infrastructures, including core networks and cell towers, have to be installed at the service area. In addition, the construction cost increases as the service area and the infrastructure density increase. For these reasons, cellular network providers usually focus on populated areas. To broaden the network service area globally, some companies tried to utilize satellite communication networks to provide public network services (e.g., Starlink [1], OneWeb [2], and Kuiper [3]). These satellite networks are composed of mega-constellation of low-Earth orbit (LEO) satellites and wireless backbones. Currently, Starlink has 1414 operational satellites which orbit as high as 550 km above the earth [1]. With the mega-constellation of satellites, the network service area can be broaden around the world, and the service quality is able to be uniform regardless of location. Because of the aforementioned reasons, the 3rd generation partnership project (3GPP) has been shown interest for integrating satellite networks with terrestrial networks [4].

The satellite network service providers expect that the network capacity they provide will be sufficient to satisfy the customer demands. However, Cartesian anticipated that Starlink would face a capacity shortfall by 2028, and over a half of total subscribers could not be provided their service sufficiently [5]. Portillo et al. estimated the total throughput of satellite network services in current state [6]. According to the estimation, Starlink’s maximum system throughput is 23.7 Tbps, with 123 ground stations and 4425 satellites. With the analysis result, the authors concluded that the number of ground stations is insufficient and could be the major limiting factor. In addition, the satellite Internet services are difficult to respond to dynamic environmental changes, such as movement of users, changes in Internet usage over time, etc. As the satellites have to keep their predefined orbit and cannot change their path dynamically, the services should have sufficient network capacity in preparation for dynamical changes. However, the limited number of orbits restricts the maximum number of operating satellites.

To improve the shortcomings of the satellite network services, we suggest an aerial access network (AAN) system where LEO satellites and high-altitude unmanned aerial vehicles (UAVs) cooperate to provide Internet services to users. The main role of high-altitude UAVs in our proposed system is to provide Internet service in areas where traffic demand is high. To provide Internet service with UAVs effectively, the system has to calculate the optimal locations where UAVs provide services to users based on the traffic demand distribution. A huge amount of computation is required for the method of calculating the optimal location points with considering the amount of traffic changing in real time and the movement of satellites. Thus, this method does not guarantee real-time performance and cannot deal with unexpected factors or situations. In comparison, the trained neural network is able to determine the next movements of UAVs by considering the situations and to take appropriate countermeasures against the changes of various factors around UAVs immediately. Thus, the proposed system utilizes federated reinforcement learning (FRL) which is a highly suitable learning method for UAV systems. Our proposed system allows UAVs to be able to consider required traffic on the ground, move to the proper locations, and provide network services autonomously. Using the system, the UAVs effectively offload the network traffic to nearby satellites which have sufficient network resources to serve.

To summarize the contributions of this paper,

- we proposed a novel AAN system design with the FRL-enabled UAV and the LEO satellites,
- we presented FRL-enabled UAVs which find areas with high traffic demand based on traffic map, and
- we validated that the UAV-aided AAN provides more network resources and has less latency than the satellite-only AAN.

This paper is organized as follows. In Section 2, we describe preliminary knowledge and studies related to our research. We explain our proposed system and give detailed explanations about the system design and learning algorithm in Section 3. After that, we explain the implementation, experiments, and performance evaluation results in Section 4. Finally, Section 5 concludes this paper with explaining remarks and future directions.

## 2. Preliminaries and Related Works

This section contains the preliminary knowledge and work related to our proposed system. We first describe AAN services then give explanations about various learning techniques.

### 2.1. Aerial Access Network

In general, Internet services are provided through core networks on the ground. In order to provide Internet services to a certain area, it is necessary to install a core network infrastructure in the area. Therefore, in rural areas where it is hard to install wired network infrastructures, it is difficult to provide the Internet service to the users in such area. In this case, the wireless backhaul could be applied instead of the wired infrastructure. The Internet service providers, including LigoWave [7], Proxim [8], and FiberLight [9], utilize wireless backhaul to provide Internet services to the customers. Furthermore, to expand the coverage and fill coverage hole, some researchers utilize UAVs as relay of wireless backhaul. Ansari et al. proposed a UAV system which can communicate with base stations to provide the Internet service to the users, where Internet service is unavailable with the wired infrastructure [10,11]. Moreover, the satellite Internet is also used in areas, such as mountains, seas, and the sky, where Internet services are not provided via ground facilities. The early stage of satellite network has quite high latency and low capacity as the network is mainly based on geostationary Earth orbit (GEO) satellites. However, compared to the past, the construction cost of satellite network was significantly reduced due to the technological progress, including the launch vehicle reuse. Thus, the satellite communication network with LEO satellites, which have quite lower orbit altitude than GEO satellites, has been proposed and deployed [12]. The LEO satellite network can provide low-latency network services due to short distance between the satellites and ground stations. Currently, Starlink [1], OneWeb [2], and Kuiper [3] are providing or preparing Internet services using LEO satellites. In the case of satellites, it costs a lot of money to launch and manage, and there are many restrictions due to the nature of orbiting the Earth. To overcome these limitations, Facebook and Boeing conducted a research on the method to use high-altitude UAVs, also known as high altitude pseudo-satellites [13,14].

As wireless communication is essential to build and maintain the satellite network, some researches focused on improving utilization of wireless communication. Sheng et al. proposed a broadband satellite network based on software-defined networking and network virtualization to achieve efficient cooperation among various resources [15]. Jia et al. proposed a channel selection optimization method based on joint cooperative spectrum sensing with cognitive radio [16]. Sharma et al. studied about satellite cognitive communications with spectrum sharing [17]. Furthermore, there were studies on the multi-layer satellite networks which utilize satellites with different orbitals, such as GEO, medium Earth orbit (MEO), and LEO. Akyildiz et al. proposed a multi-layered satellite routing algorithm which effectively utilizes the delay measurements [18]. In addition, many researchers tried to integrate terrestrial networks and satellite networks [19]. A research on satellite Internet of things (IoT) was also conducted to obtain the connectivity of IoT devices using satellite networks. Unlike the above researches, Portillo et al. suggested that the bottleneck of satellite networks is due to the limited capacity of communication between satellites and ground stations [6]. Furthermore, Cartesian emphasized that, due to the nature of satellite, there are bound to be problems related to the congestion in user-dense areas [5].

In summary, the existing researches about the satellite-based AAN mainly focused on wireless communication, such as cognitive radio, spectrum sensing, channel selection, and so on. Furthermore, the previous studies on AAN with UAVs did not actively consider the satellite network as their backbone.

Motivated by this, in this paper, we propose a FRL-based system which utilizes pseudo-satellites for supporting the satellite communication network and increasing the capacity of network service.

### 2.2. Deep Reinforcement Learning

Reinforcement learning (RL) is a mathematical framework for computing devices to perform learning autonomously based on experience, and the core of RL is learning through interactions with the environment [20,21]. In RL, an agent selects an action using the policy, that is the basis of selecting the action, and performs the chosen action in the environment. Then, the agent observes the state of the changing environment and obtains the rewards from the environment. By repeating the above process, the agent continues to update the policy so that a better action can be selected. The best order of actions is determined by the rewards provided by the environment, and the purpose of the agent is to learn the optimal policy that maximizes the expected compensation values. The algorithm or mechanism for the agent to perform learning depends on the RL method.

In relatively simple environments, it is possible to define all states that an agent can have. However, for complex problems, it is hard to predict all states and to consider all possible actions in each state. To overcome this limitation, deep reinforcement learning (DRL) was defined by applying deep neural networks to existing reinforcement learning [22]. The DRL trains the natural network based on the state and reward resulted from the action to find the optimal action based on the state. In recent years, various DRL algorithms have been proposed, and there many DRL algorithms, such as Deep Q network (DQN) [23], Deep Detergent Policy Gradient (DDPG) [24], Asynchronous Advantage Actor–Critic (A3C) [25], Trust Region Policy Optimization (TRPO) [26], Proximal Policy Optimization (PPO) [27], Soft Actor–Critic (SAC) [28], etc.

### 2.3. Federated Learning

Without data, any learning in artificial intelligence cannot be done. In most cases, these data are distributed, so the most general solution is to centralize these data and then perform learning on the central server. However, it is not easy to gather much of the data needed for learning in one place, as it requires much computing and communication resources. Unlike conventional centralized learning approaches, federated learning (FL) is a model in which devices in different spaces perform their own learning and work with other devices to form the global learning model. The concept of FL was introduced by Google in 2016 and FL has been applied to Google keyboard, Gboard [29]. FL can be applied to all edge devices, so FL has great potential to be applied in a variety of fields [30]. In the beginning of FL process, the server shares a common model with participants. After that, each participant trains the model they received from the server based on their own data and sends local model parameters back to the server. The server integrates the collected local model parameters to build a global model, and shares it with participants again. The above operations are repeated to get the well-trained global model.

It is not easy for UAVs to have stable and reliable connectivity and abundant communication resources due to high mobility of UAVs and wireless communications, Thus, in UAV systems, it is difficult to reliably transfer the data used for learning to a central server every time, so conventional learning methods do not work well with a limited amount of data in a real time. A UAV has a computational ability and resources to perform learning independently. By utilizing FL, each UAV trains its own model based on its own data and exchanges only model parameters, so FL is highly suitable for UAV systems. Furthermore, as different data from many UAVs are utilized together to build a global model, a much better trained model can be obtained than when a single UAV performs independent learning with only its data. Consequently, FL can be well applied to and effective in UAV systems [31].

### 2.4. Federated Reinforcement Learning

FRL, a combination of FL and RL, was first introduced in [32]. In this study, the authors showed that FRL techniques can utilize observations from various environments together for RL. Furthermore, the study verified that FRL performs better than general DQNs based on partial observations of the same environment. FRL are applied to various technical fields including autonomous driving and robot system control. As explained in Section 2.3, FL can be applied to UAV systems well and effectively. Thus, applying FRL to UAV systems can make comprehensive use of various observations collected from different environments with performing efficient communication, which allows UAVs to learn better on their own. Motivated by this, we proposed the system which utilizes FRL for UAVs to be able to consider required traffic on the ground, move to more proper locations, and provide network services autonomously.

## 3. System Design

In order to give flexibility and enhance coverage expansion to the existing terrestrial communication network, we suggest FRL-based AAN, composed of LEO satellites and UAVs, which could be adaptable to various changing communication network environments. In this section, we first explain the overall design and operations in the proposed system. After that, we give a detailed explanation about the proposed system, including the learning method used in the proposed system.

### 3.1. System Concept

**Figure 1:**
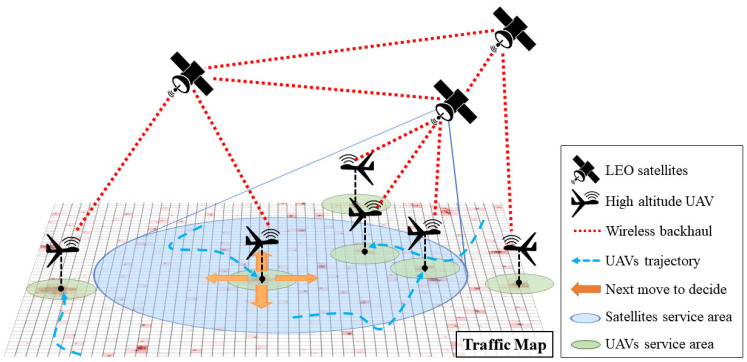
The concept of the proposed system.

Figure 1 shows the concept of the proposed system. The proposed system includes the backbone based on LEO satellites and FRL-enabled UAVs, and provides public network services. In the satellite communication system, the satellites cover wide area to provide services to many devices with limited communication resources. Due to the satellites’ limited resources, it is difficult for the satellite communication system to provide services to all wireless devices in areas with dense traffic demands. To increase the communication resources, UAVs are deployed to the areas with dense traffic and they provide network services to the devices which receive communication services with insufficient resource from the satellites. Each UAV takes off from its initial location and then autonomously and independently changes its position by considering the traffic distribution on the ground. Then, the UAVs properly perform the data routing to satellites by considering the service capability of satellites and the distance from them. The UAVs determine their movement based on their own neural network which is trained using our FRL algorithm. A huge amount of computation is required for the method of calculating the optimal location points with considering the amount of traffic changing in real time and the movement of satellites. Thus, this method does not guarantee real-time performance. In addition, such method cannot deal with unexpected factors or situations. In comparison, using the trained neural network, it is possible to determine the next movements of UAVs by considering the situations and to take appropriate countermeasures against the changes of various factors around UAVs immediately.

### 3.2. Federated Reinforcement Learning System

**Figure 2:**
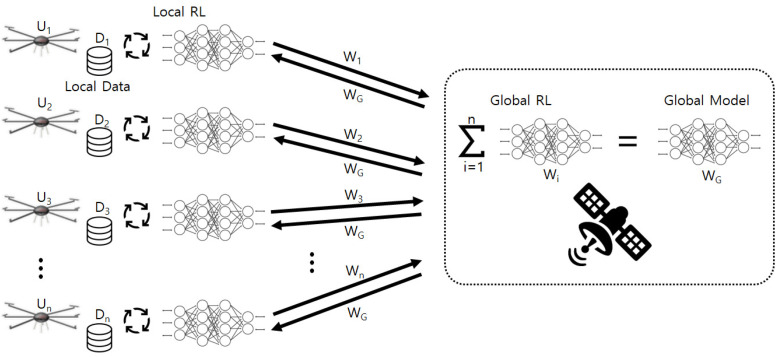
The overall operations of FRL in the proposed system.

As explained before, we utilized FRL to train UAVs’ neural network, and Figure 2 shows the overall operations of FRL in the proposed system. To explain the FRL scheme for our system, we assume that there are *n* UAVs, $U_{1}$, ⋯, $U_{n}$, which have their own databases, $D_{1}$, ⋯, $D_{n}$. The FRL in the system includes the following major steps. First, a server, a satellite in our system, sends the initial global model to all of the UAVs, and then each UAV trains its local model using local information including states, actions, and rewards. After that, the UAVs send local model parameters, $W_{1}$, ⋯, $W_{n}$, back to the server, and the model parameters are aggregated into the global model in the server. The parameters of the aggregated global model, $W_{G}$, are delivered to the UAVs again, and the above procedures are repeated until the global model is trained enough.

### 3.3. Reinforcement Learning Algorithm

In this subsection, we explain the RL algorithm used in the proposed system. The PPO algorithm is based on the actor–critic concept which uses two separate networks [27]. The actor network determines the optimal behavior of an agent, while the critic network evaluates the policy and trains the actor using rewards. PPO was inspired by TRPO, and PPO provides a more direct approach to implementing and coordinating tasks for learning in comparison with TRPO. Furthermore, PPO is known to provide simpler and superior performance than TRPO in many areas [33]. Thus, the PPO algorithm is suitable for various tasks performed by UAVs in the context of UAV system control because of the algorithm’s short calculation time [34]. Actually, many researches on UAVs utilized the PPO as RL algorithms, and many results showed that the PPO outperforms other algorithms in various UAV operations, such as attitude control, landing, waypoint navigation, etc [34]. Thus, we selected the PPO as a learning algorithm for our system.

### 3.4. Environment Configuration

The agent performs learning by interacting with the environment. Thus, we constructed the environment for the agent to perform the learning properly, so that the UAVs with the trained neural network are able to perform the missions explained in Section 3.1. This section gives a detailed explanation about the environment configuration, including the traffic map, the state, the action, and the reward.

#### 3.4.1. Traffic Map

**Figure 3:**
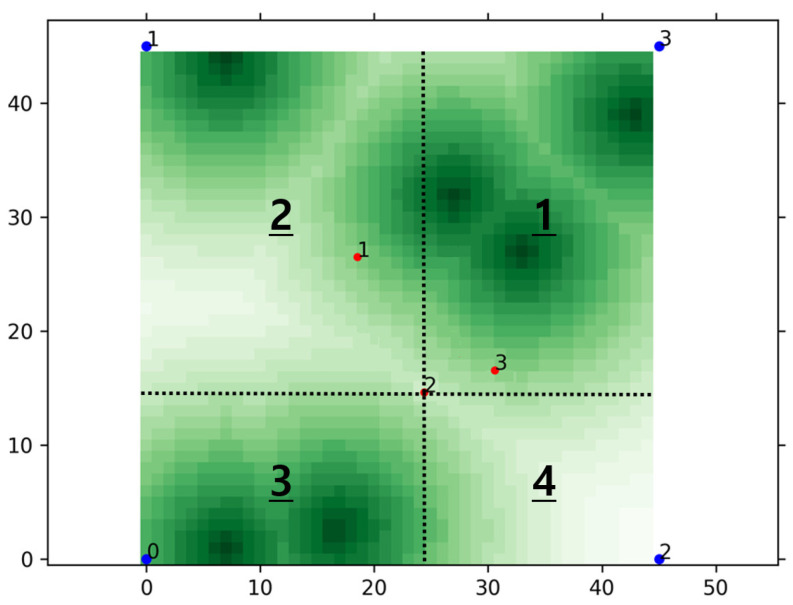
An example of network traffic map.

In the scenario described in Section 3.1, UAVs should move to appropriate locations and provide network services autonomously. In order to do this, we built various network environments with different traffic distributions for the agent to perform learning from diverse experience. Figure 3 shows an example network traffic map, and darker color means higher traffic on the ground. In the figure, red and blue points with a number mean UAVs and satellites, respectively. We set the traffic map to periodically change the traffic distribution whenever a certain number of episodes are finished, during the training process. By doing so, the agent can perform learning in various environments, which alleviates any bias which can be formed from a specific environment.

#### 3.4.2. State

In order for an agent to perform an optimal action suitable for the situation, the state should include appropriate information. We designed the state in the environment to be composed of 4 value sets, and each set has 4 values. Algorithm 1 shows the pseudocode for getting the state, and Table 1 lists the variables used in Algorithm 1.
**Algorithm 1** Algorithm for getting the state1:**for** each UAV *u* in U **do**2:   **if** *u* is not the chosen drone **then**3:     **if** u.x < x **then** N[0] += 14:     **if** u.x > x **then** N[1] += 15:     **if** u.y < y **then** N[2] += 16:     **if** u.y > y **then** N[3] += 17:   **end if**8:**end for**9:T${}_{o}$[0] = sum(sum(M${}_{o}$[0:x+1,0:v]))10:T${}_{o}$[1] = sum(sum(M${}_{o}$[x:h,0:v]))11:T${}_{o}$[2] = sum(sum(M${}_{o}$[0:h,0:y+1]))12:T${}_{o}$[3] = sum(sum(M${}_{o}$[0:h,y:v]))13:temp = sum(T${}_{o}$)14:**for** i in range(0,4) **do**15:   S[i] = T${}_{o}$[i] / temp16:**end for**17:**for** i in range(4,8) **do**18:   S[i] = T${}_{o}$[i-4] / N[i]19:**end for**20:temp = sum(S[4:8])21:**for** i in range(4,8) **do**22:   S[i] = S[i] / temp23:**end for**24:T${}_{r}$[0] = sum(sum(M${}_{r}$[0:x+1,0:v]))25:T${}_{r}$[1] = sum(sum(M${}_{r}$[x:h,0:v]))26:T${}_{r}$[2] = sum(sum(M${}_{r}$[0:h,0:y+1]))27:T${}_{r}$[3] = sum(sum(M${}_{r}$[0:h,y:v]))28:temp = sum(T${}_{r}$)29:**for** i in range(8,12) **do**30:   S[i] = T${}_{r}$[i-8] / temp31:**end for**32:**for** i in range(12,16) **do**33:   S[i] = T${}_{r}$[i-12] / N[i]34:**end for**35:temp = sum(S[12:16])36:**for** i in range(12,16) **do**37:   S[i] = S[i] / temp38:**end for**39:**return** S

In the scenario, it is reasonable for the UAV to provide a network service located on an area with a lot of traffic. Therefore, we designed the first value set to include traffic information, and the first value set in the state is composed of traffic sum values on the agent’s left, right, bottom, or top side. For example, in Figure 3, the traffic sum value on the left side of UAV 2 is the sum of traffic in the regions indicated by the numbers 2 and 3 with an underline. Lines 9 to 16 in Algorithm 1 are relevant to these operations. Lines 13 to 16 are required to normalize the traffic values for better learning.

As explained in the above, it is reasonable for UAVs to move to locations with a lot of traffic. However, it is inappropriate for all the UAVs to flock to one location because they cannot provide services to a wide area. Therefore, in addition to the traffic information, relative locations of neighboring UAVs should also be considered. The second set is composed of the values for taking into account the traffic and the positions of surrounding UAVs together. The fifth to eighth values of the state are the traffic sum value divided by the number of neighboring UAVs on the left, right, bottom, or top of the UAV, respectively. For example, the first value of the second set is the value obtained by dividing the sum of traffic in the regions by the number of neighboring UAVs on the left side of the UAV. Lines 1 to 8 and 17 to 23 in Algorithm 1 are relevant to these operations.

**Figure 4:**
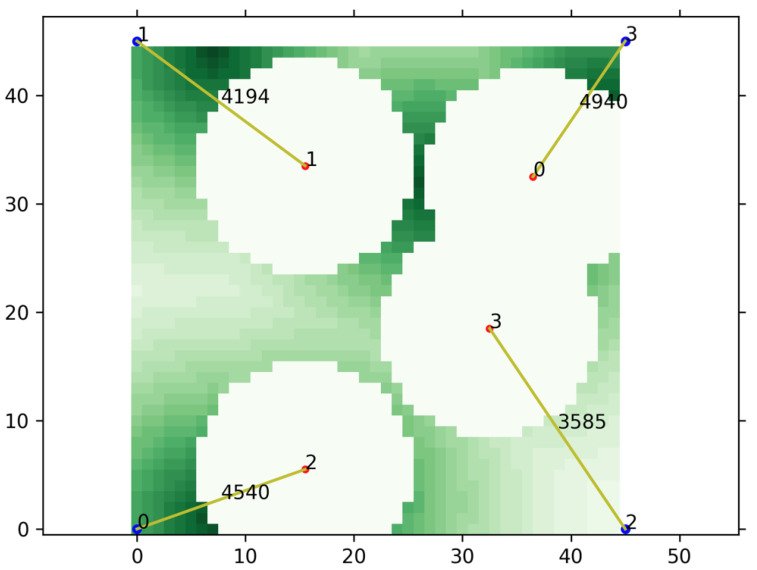
An example of providing network services to the ground.

Until now, we considered traffic on the ground without taking account of the network services provided by UAVs. Unlike Figure 3, Figure 4 shows an example of UAVs providing data communications to the ground in the assumed scenario. As shown in the figure, each UAV provides a network service for traffic on the ground around one’s location. The white part in the figure means an area where there is no traffic left because of UAVs providing network services. The yellow lines indicate communications between the UAVs and satellites, and the number on the lines shows the amount of transmission. The most ideal result is that UAVs are well placed to minimize the sum of the demanded traffic remaining in the traffic map. Thus, unlike the sets 1 and 2, we designed the sets 3 and 4 to contain information about the remaining traffic. Similar to the set 1, the set 3 is composed of the sum values of the remaining traffic on the UAV’s left, right, bottom, or top side. Similar to the set 2, the set 4 is composed of the remaining traffic sum value divided by the number of UAVs on the left, right, bottom, or top of the UAV, respectively. Lines 24 to 38 in Algorithm 1 are relevant to these operations.

#### 3.4.3. Action

In order to conduct reliable communications with satellites and eliminate potential risks to obstacles such as buildings, it is assumed that UAVs maintain their altitude for stable flight. Therefore, the actions of UAVs simply include moving in four directions, left, right, bottom, and up on the traffic map. In other words, UAVs can move east, west, south, or north in the real world as shown by the yellow arrows in Figure 1. In addition to these 4 directions, a UAV has a total of five actions by adding one more action, staying in the position.

#### 3.4.4. Reward

In order for an agent to perform well in learning, appropriate rewards must be given. Algorithm 2 shows the details of the reward determination procedure.
**Algorithm 2** Algorithm for determining the reward value1:remained_traffic${}_{t}$= **CalRemTraff** (M${}_{r}$)2:**MoveUAV** (action)3:remained_traffic${}_{t+1}$= **CalRemTraff** (M${}_{r}$)4:reward = remained_traffic${}_{t}$− remained_traffic${}_{t+1}$5:**if** reward < 0 **then**6:   reward = −0.57:**else if** reward == 0 **then**8:   **if** action == ‘staying’ **then**9:     **if** A UAV consumes all of its network capability **then**10:        reward = 0.511:     **else**12:        reward = −213:     **end if**14:   **else**15:     reward = 0.516:   **end if**17:**else**18:   reward = 219:**end if**

In the algorithm, a function, CalRemTraff, calculates the remained traffic in the traffic map, M${}_{r}$. The other function, MoveUAV, makes a UAV move according to the input action, which forwards the step of episode in a learning process. As shown in Figure 4, in the assumed scenario, each UAV provides a network service for traffic on the ground around one’s location. The most ideal result is that UAVs are well placed to minimize the sum of the required traffic remaining in the traffic map. Therefore, it is reasonable to receive a reward if the remaining traffic decreases as the UAV moves, and line 18 in Algorithm 2 indicates this situation. Conversely, if the remaining traffic increases due to the movement of the UAV, the penalty, the negative reward, should be given, and lines 5 and 6 are relevant to these operations.

In addition to the above situations when the remaining traffic increases or not, there is also a situation in which there is no change in the remaining traffic, and we divide this situation into two cases. The first case is when the UAV stays in the position without moving, and we again divide this case into two sub-cases. The first sub-case is when a UAV has already consumed all of its network service capability. In this sub-case, it is a reasonable to continue to provide services with maintaining its position because it makes the most of the UAV’s capability, so the UAV receives a reward as indicated by lines 9 to 10 in Algorithm 2. The second sub-case is a situation in which a UAV does not consume all of its capability, which means that it can provide network services more. In this sub-case, staying in the position can be abandoning the opportunity for movement to explore a better position, so the UAV receives a negative reward. Lines 11 and 12 are relevant to these operations. The second case is when a UAV moves, but the remaining traffic does not change. In this case, although the remaining traffic is not decreased, the environment gives a positive reward to the UAV to encourage the UAV to move for finding a better location. Lines 14 and 15 in Algorithm 2 are relevant to these operations.

## 4. Performance Evaluation

In this section, we give detailed descriptions about two key implementations—FRL and network simulator implementations—for evaluations of the proposed system. Furthermore, we explain various experiments and show the evaluation results that the UAV-aided AAN could operate efficiently in densely populated areas where the satellites cannot provide sufficient Internet services, which improves network performances.

### 4.1. Federated Reinforcement Learning

In this subsection, we give detailed explanations about the learning for the proposed system. We describe the implementation, the learning process, and the simple evaluation for validating the result of learning.

#### 4.1.1. FRL Implementation

For performance evaluations, we constructed the RL model of the proposed system utilizing PyTorch library [35] referring to the work in [36]. Table 2 shows the hyper parameters used in the PPO algorithm. Using the RL model, we additionally implemented the FL, referring to the work in [37], to build our proposed FRL system. We built the system on Ubuntu 20.04 using the desktop equipped with AMD Ryzen™ 7 5800X and 32GB RAM. We trained the learning model by utilizing NVIDIA’s compute unified device architecture (CUDA) on NVIDIA GeForce RTX 3070 8GB GDDR6 PCI Express 4.0 graphic card for faster learning. We constructed traffic map, shown in Figure 3, referring to 2D Gaussian grid map introduced in [38].

#### 4.1.2. Learning Process

As explained in Section 3.2, Section 3.3, Section 3.4, we performed FRL on the implementation described in Section 4.1.1. Episode is a unit of learning, and each episode ends after a certain number of steps forward. At the end of the episode, we records the sum of the reward values obtained by the agent in the episode as the score of the episode. We trained the agent with periodically changing the traffic distribution on the map whenever a certain number of episodes are finished as explained in Section 3.4.1. Thus, the agents were able to perform learning in various environments, alleviating any bias which can be formed from a specific environment.

**Figure 5:**
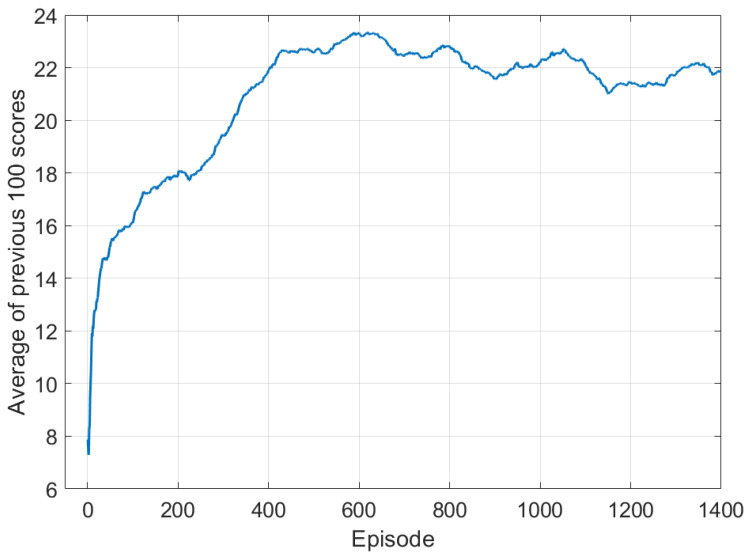
Average value of scores as the episode passes.

In the learning process, the agent continues to update the policy so that a better action can be selected, and the purpose of the agent is to learn a policy that maximizes the expected compensation values. Thus, we monitored the sum of the scores obtained in the last 100 episodes during learning, and Figure 5 shows the result. As shown in the figure, the average of score values increases as the episode passes, which means that the more learning were repeated, the better the agent performed the mission. The average value continues to increase up to about 600 episodes and reaches the saturation, so we decided to perform learning until 1000 episodes.

**Figure 6:**
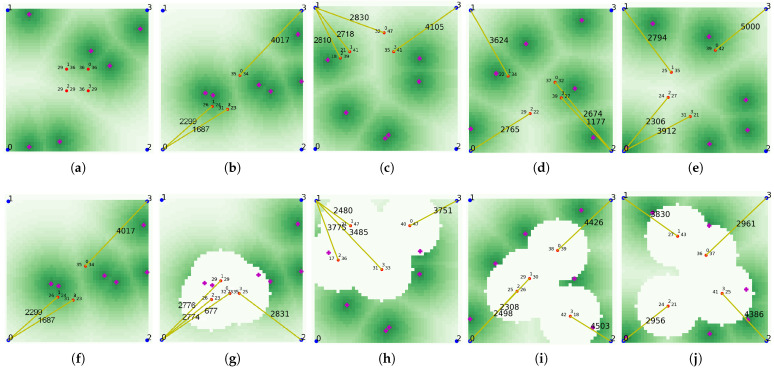
Traffic map and UAV deployment as the episode passes. (**a**) 0 episode—traffic map. (**b**) 100 episode—traffic map. (**c**) 200 episode—traffic map. (**d**) 600 episode—traffic map. (**e**) 1000 episode—traffic map. (**f**) 0 episode—deployment. (**g**) 100 episode—deployment. (**h**) 200 episode—deployment. (**i**) 600 episode—deployment. (**j**) 1000 episode—deployment.

We periodically conducted an evaluation where UAVs make their own judgments in consideration of traffic and move according to policy based on the neural network being trained during performing learning. In Figure 6a–e, in the upper row shows the traffic map of each episode, and the lower subfigures, Figure 6f–j, show the final deployment of UAVs in each episode. As shown in the figure, in episode 0 where learning was not performed at all, UAVs are located close to each other and do not respond appropriately to traffic. This phenomenon can also be seen in the case of episode 100, where learning was not sufficiently performed. However, as the episode proceeds and the agent performs learning, UAVs are located depending on the distribution of traffic as shown in results of episodes 200, 600, and 1000. In addition, UAVs spread appropriately without clumping together to provide network services in a wide range.

#### 4.1.3. Validation of Learning Result

**Figure 7:**
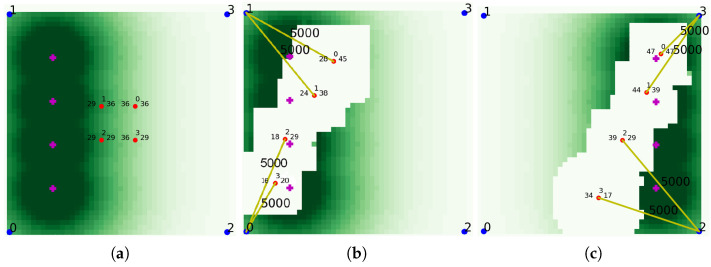
Traffic map and UAV deployment of the simple evaluation. (**a**) Initial situation. (**b**) Left side traffic situation. (**c**) Right side traffic situation.

In order to validate the result of learning, we performed a evaluation using the trained network, and Figure 7a shows the initial state of this experiment. As shown in the Figure 7a, UAVs are initially placed in the middle and traffic is distributed on the left side of the map. After sufficient steps proceeded to give UAVs enough time to move to the desired location as shown in Figure 7b, the distribution changes to place traffic on the right side of the map as shown in Figure 7c. Again, after predetermined steps to give UAVs enough time to change their location, the traffic is rearranged back to the left side of the map, and the above processes were repeated several times. As shown in the figures, the UAVs recognized the traffic distribution and moved to the left, and then the UAVs moved to the right as the traffic distribution changed. The video of the experiment can be found at [39]. As shown in the figures and video, we can see that UAVs quickly deal with the change of traffic distribution and are properly deployed without clumping together to provide network services intelligently. As explained before, the number on the lines shows the amount of transmission. As shown in the figure, UAVs provided communication services using all of their service capability which was set to 5000 in this evaluation, which means that the UAVs were in proper locations.

### 4.2. Evaluate System with Network Simulator

To evaluate the overall design of our proposed system, we built our own simulation for evaluating network performance of AAN. The simulation was built on network simulator 3 (NS3)-based [40] LEO satellite network simulation framework [41], which does not include UAVs in the simulation environment. To build our own simulation, we implemented functionalities related to UAVs and connections between satellites and UAVs with inter satellite links (ISLs). Furthermore, our simulator receives traffic map which represents traffic demand and the position of the UAVs as input.

#### 4.2.1. Network Simulator Implementation

**Figure 8:**
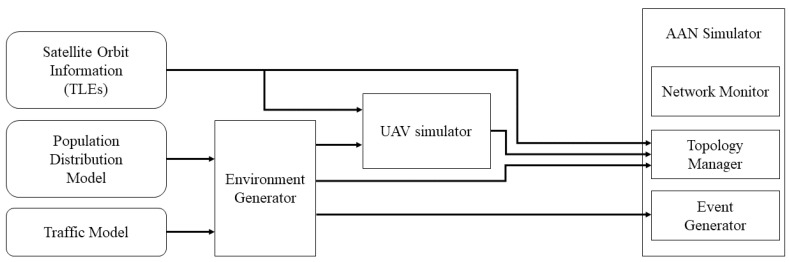
The overall structure of AAN simulator.

For system evaluations, we built the network simulator, and Figure 8 represents the structure of the simulator. The simulator consists of three components, simulation environment generator, UAV simulator, and AAN simulator. The environment generator receives environmental information, including user distribution and traffic model, and generates traffic map. The UAV simulator simulates UAVs’ position, mobility, and communication with considering forwarding table and data rate information. The AAN simulator conducts network simulation whose environment is composed of satellites, UAVs, and user nodes. The simulator generates access a network topology using the information about satellites and UAVs, and creates events based on traffic map. The components of AAN are highly mobile and the network topology changes continuously. Thus, during the simulation running, the simulator continuously updates the network topology and connectivity information based on the updated positions of satellites and UAVs.

#### 4.2.2. Configuration

To evaluate our proposed system, we designed the simulation which aims to measure the total amount of throughput which the AAN or satellite-only network could serve. In the simulation environment, every consumer sends data to one sink node through the non-terrestrial network. We adopted Starlink satellite model [1] as our satellite network model. Furthermore, we adopted the high altitude pseudo-satellite model of Facebook’s aquila [13], where satellites could operate at 27 km altitude, as our high altitude UAV model. In addition, the UAVs are equipped with a device capable of ground-satellite link (GSL) and inter-satellite link (ISL) communication. Similar to the Starlink network, we configured the simulation where UAVs are equipped with communication equipment equivalent to that of Starlinks satellites and the UAVs’ operating altitude is 27 km. With this assumption, we configured that the satellites’ the ratio of the service area to operating altitude is the same as that of the UAVs. For this reason, we set our UAVs’ service area radius to 28.2 km because the satellites’ operating altitude is 550 km and the radius of service area is 573.5 km. The detailed simulation configuration is represented at Table 3.

**Figure 9:**
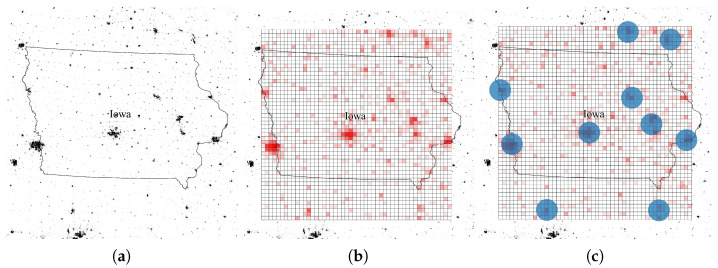
Simulation environment map with population distribution and UAVs’ position. (**a**) Raw population distribution. (**b**) Processed population distribution. (**c**) UAVs’ position.

We selected Iowa State of United States as the simulation area based on population distribution. The state has several densely populated cities and many small towns, so we choose the states as simulation area. The population distribution is represented in Figure 9a. In the figure, darker and lighter points represent the area with dense and sparse population, respectively. For traffic model, we utilized the monthly data traffic per smartphone according to the Ericsson Mobility Report [42] and daily traffic usage pattern model referring to [43]. Based on the traffic model, total required data rate of the service area is 313.6 Gbps. However, as not all the smartphone users use satellite Internet, we assumed that 5% of them use the satellite Internet. Therefore, we configured the total traffic demand rate of entire environment as ~15.7 Gbps. Within the traffic model, we generate traffic map based on the population distribution according to the work in [44], which is represented at Figure 9b. In this figure, the intensity of red color represents the degree of traffic demand. Figure 9c shows UAVs’ position in the simulation. We deployed 10 UAVs to provide more Internet service resources to the service area. Through out the simulation, UAVs were located at the center points of each circle and provided Internet service to the users inside of the circle.

#### 4.2.3. Simulation Result

**Figure 10:**
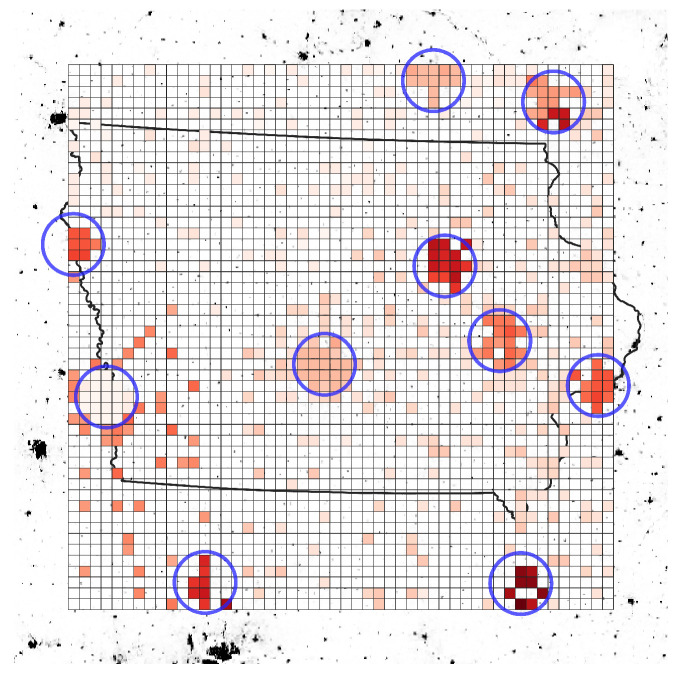
Simulation environment map with throughput improvement rate.

In our proposed AAN, the UAVs are deployed to provide network resource to the areas with high network traffic demand. The overall transmitted data size is related to the Internet service capacity of AAN. Therefore, to validate our system with UAVs, we measured the total amount of data transmitted by customer nodes throughout the simulation. We compared the transmitted data size of customer nodes with two types of AANs, satellite-only AAN and UAV-aided AAN. As a result, our proposed UAV-aided AAN provides approximately 3.25 times more communication resources than satellite-only AAN, and Figure 10 represents throughput improvement rate of each area. In the figure, the intensity of color represents the improvement rate of each area, and blue circles represent service ranges of deployed UAVs. As shown in the simulation result, using the proposed system, it is possible to deliver more data from densely populated area. Moreover, the traffic demand of densely populated area can be offloaded to UAVs, so the satellites can provide more resources to rural areas, which increases the throughput of rural areas.

In addition to the throughput improvement comparison, we compared the network path between the simulations with satellite-only AAN and UAV-aided AAN.

**Figure 11:**
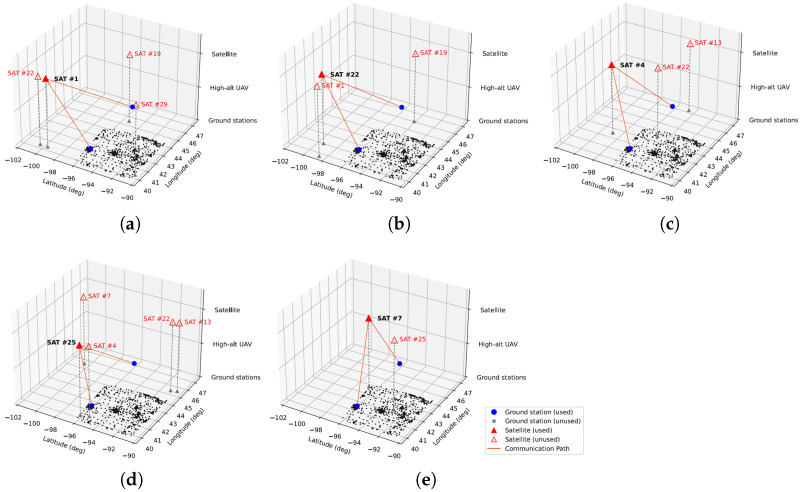
Network path of the satellite-only AAN. (**a**) t = 0.0 s. (**b**) t = 17.0 s. (**c**) t = 85.8 s. (**d**) t = 140.3 s. (**e**) t = 191.9 s.

**Figure 12:**
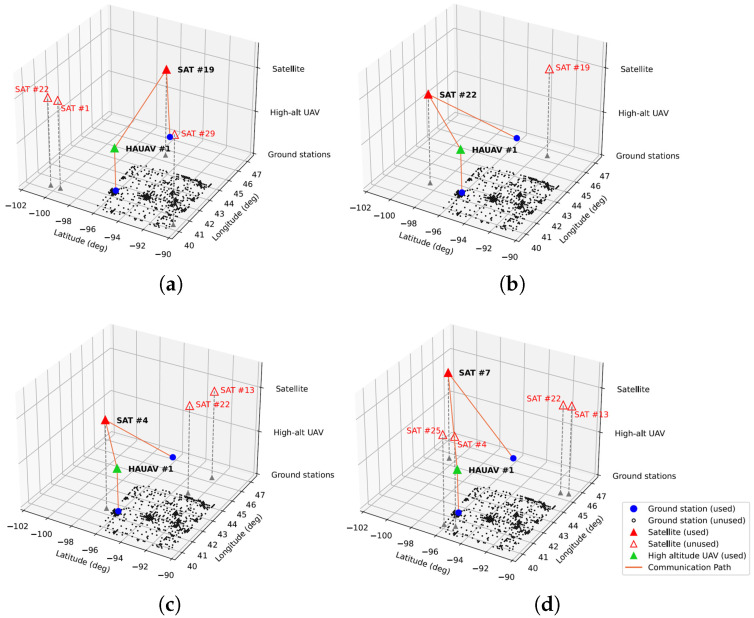
Network path of the UAV-aided AAN. (**a**) t = 0.0 s. (**b**) t = 28.9 s. (**c**) t = 114.2 s. (**d**) t = 140.5 s.

The network path of satellite-only AAN is represented at Figure 11, and Figure 12 shows the path of UAV-aided AAN. Throughout the simulation, the path of satellite-only AAN changed four times, at 17.0, 85.8, 140.3, and 191.9 s. The initial network path of satellite-only AAN is represented at Figure 11a, and changed network paths are represented at Figure 11b,c and Figure 12d,e. By contrast, the path of UAV-aided AAN changed three times, at 28.9, 114.2, and 140.5 s, which is less than satellite-only AAN. The initial network path of UAV-aided AAN is represented at Figure 12a, and changed network paths are represented at Figure 12b–d. As we can see the difference between the two cases, the UAVs could offload the traffic of densely populated areas, alleviate the bottleneck of the network link between satellites and ground nodes, and increase the total network capacity of the AAN. Furthermore, the UAV-aided AAN has the fewer number of network path changes which could adversely affect communication status, such as temporary communication disconnection, communication re-initialization, etc.

**Figure 13:**
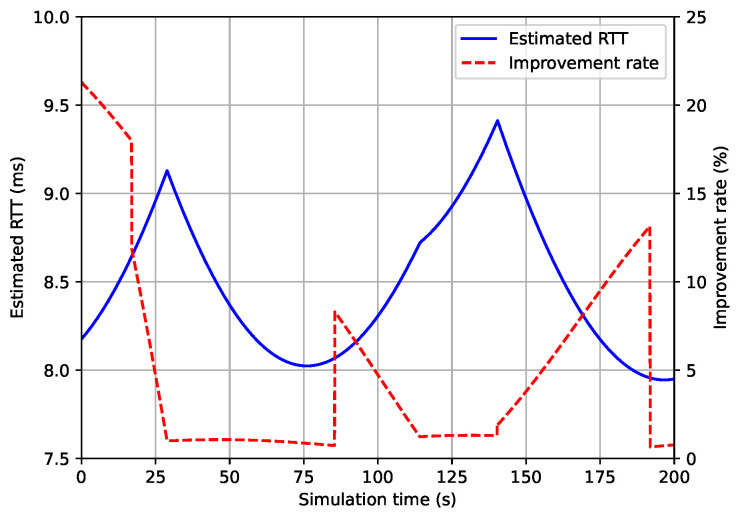
Round trip time and it’s improvement rate of UAV-aided AAN w.r.t satellite-only AAN.

Based on the network paths of satellite-only and UAV-aided AAN, we compared the round trip time (RTT) of each network path. The comparison result is represented at Figure 13. In the figure, the solid blue line represents the estimated RTT based on UAV-aided AAN’s network path, and the red dotted line represents the improvement rate of UAV-aided AAN in comparison with satellite-only AAN. As the result, the RTT of UAV-aided AAN is less than up to 9.5 ms and has 5.1% of improvement rate in average compared to the RTT of satellite-only AAN.

## 5. Conclusions

In this paper, we proposed AAN with LEO satellites and high-altitude UAVs equipped with FRL techniques. By utilizing FRL-based UAVs, which could determine their next destination based on the collected various information, our proposed system automatically detects the area where communication resources are scarce based on the network traffic map. Moreover, the UAVs improve itself continuously without the director’s guidance, and the system could respond to changing environment without further configuration change. We evaluated our proposed system with traffic map simulation and network simulator. Based on the evaluation result, we showed that the proposed system could provide network service in various area, including the area where network resource demand distribution rapidly changes. In the evaluations, the UAVs could communicate with the satellites and the terrestrial devices, so the UAVs could process the network traffic load in densely populated area, which could alleviate the load of the satellites. With this feature, the UAV-aided AAN enhanced the throughput of communications between the ground devices and the satellites and shorten the path length between them. As a result, our proposed AAN provided 3.25 times more communication resources and had 5.1% lower latency than satellite-only AAN. Moreover, the UAV-aided AAN had fewer network path changes than the satellite-only AAN, which provided more stable Internet services to the users.

As a future work, we will utilize more various simulators to consider more environmental factors including the network characteristics, population shift model, and the network traffic demand model. Furthermore, we will analyze more various indicators representing the performance of the AAN and satellite Internet service. Moreover, we plan to integrate our proposed system with terrestrial network, which has abundant but difficult network resources to be shared with areas far from the terrestrial network area.

## Figures and Tables

**Table 1:** Variables used in Algorithm 1.

Notation	Description
M${}_{o}$	Original traffic map.
M${}_{r}$	Remained traffic map.
U	List of all UAV objects.
N	List of the number of neighboring UAVs.
S	List of state.
n	The number of UAVs
x	the chosen UAV’s *x* position
y	the chosen UAV’s *y* position
h	The horizontal length of traffic map
v	The vertical length of traffic map

**Table 2:** Hyperparameters and values used for learning.

Hyperparameter	Value
Horizon value	20
Minibatch size	5
Number of epochs	4
Learning rate	0.0003
Generalized advantage estimator	0.95
Clipping parameter	0.2
Discount factor gamma	0.99
Value function coefficient	0.5
Optimizer algorithm	Adam
Critic network dimension	16 × 256 × 256 × 256 × 5
Actor network dimension	16 × 256 × 256 × 256 × 5

**Table 3:** Aerial access network simulation configuration.

Simulation Configuration	Value
Satellite operating altitude (km)	550.0
Satellite service area radius (km)	573.5
High-alt UAV operating altitude (km)	27.0
High-alt UAV service area radius (km)	28.2
Maximum ISL range (km)	5016.6
Maximum GSL range (km)	794.6
Maximum ISL rate (Gbps)	10.24
Maximum GSL rate (Mbps)	720.0

## Data Availability

Data sharing not applicable.

## Abbreviations

The following abbreviations are used in this manuscript:

AAN	Aerial Access Network
GEO	Geostationary Earth Orbit
MEO	Medium Earth Orbit
LEO	Low-Earth Orbit
UAV	Unmanned Aerial Vehicle
6G	6th Generation
3GPP	3rd Generation Partnership Project
IoT	Internet of Things
RL	Reinforcement Learning
DRL	Deep Reinforcement Learning
FL	Federated Learning
FRL	Federated Reinforcement Learning
DQN	Deep Q Network
DDPG	Deep Detergent Policy Gradient
A3C	Asynchronous Advantage Actor-Critic
TRPO	Trust Region Policy Optimization
PPO	Proximal Policy Optimization
SAC	Soft Actor-Critic
ISL	Inter-Satellite Link
GSL	Ground-Satellite Link
NS3	Network Simulator 3
RTT	Round Trip Time
